# Nationwide public perceptions regarding the acceptance of using wastewater for community health monitoring in the United States

**DOI:** 10.1371/journal.pone.0275075

**Published:** 2022-10-11

**Authors:** A. Scott LaJoie, Rochelle H. Holm, Lauren B. Anderson, Heather D. Ness, Ted Smith

**Affiliations:** 1 Department of Health Promotion and Behavioral Sciences, School of Public Health and Information Sciences, University of Louisville, Louisville, Kentucky, United States of America; 2 Christina Lee Brown Envirome Institute, School of Medicine, University of Louisville, Louisville, Kentucky, United States of America; Georgia Southern University, UNITED STATES

## Abstract

To assess the levels of infection across communities during the coronavirus disease 2019 pandemic, researchers have measured severe acute respiratory syndrome coronavirus 2 RNA in feces dissolved in sewer water. This activity is colloquially known as sewer monitoring and is referred to as wastewater-based epidemiology in academic settings. Although global ethical principles have been described, sewer monitoring is unregulated for health privacy protection when used for public health surveillance in the United States. This study used Qualtrics XM, a national research panel provider, to recruit participants to answer an online survey. Respondents (N = 3,083) answered questions about their knowledge, perceptions of what is to be monitored, where monitoring should occur, and privacy concerns related to sewer monitoring as a public health surveillance tool. Furthermore, a privacy attitude questionnaire was used to assess the general privacy boundaries of respondents. Participants were more likely to support monitoring for diseases (92%), environmental toxins (92%), and terrorist threats (88%; e.g., anthrax). Two-third of the respondents endorsed no prohibition on location sampling scale (e.g., monitoring single residence to entire community was acceptable); the most common location category respondents wanted to prohibit sampling was at personal residences. Sewer monitoring is an emerging technology, and our study sheds light on perceptions that could benefit from educational programs in areas where public acceptance is comparatively lower. Respondents clearly communicated guard rails for sewer monitoring, and public opinion should inform future policy, application, and regulation measures.

## Introduction

Sewer monitoring for public health is a tool that detects biological and chemical targets in sewage from community or institutional settings prior to treatment. This involves collecting samples from existing piped wastewater infrastructure (sewers), and this method, which is also referred to as wastewater-based epidemiology (WBE), has been deployed for a range of public health inquiries, including tracking enteroviruses [1], illicit drugs [2–4], alcohol consumption [4, 5], tobacco use [4, 6], dietary patterns [4], and biological agents as weapons [7]. During the coronavirus disease 2019 (COVID-19) pandemic, researchers measured severe acute respiratory syndrome coronavirus 2 (SARS-CoV-2) RNA in feces dissolved in sewer water to assess the levels of infection across communities [8–10]. Sewer monitoring has emerged as a potential component of innovative and cost-effective public health surveillance. However, surveillance activities can evoke privacy concerns and possible stigmatization of institutional settings or communities where high levels of health risks are identified. Current sewer monitoring methods do not involve studying human DNA markers that may be present in sewage; therefore, public debate on discarded DNA [11] is not currently applied here.

Sewer monitoring is not regulated for health privacy protection in the United States, although global ethical principles have been described based on the premise that samples are typically collected by, or with the permission of, a wastewater utility operating through publicly owned infrastructure [12–16]. The premise is that samples are anonymous and therefore informed and voluntary consent to participate in sewer monitoring is not required from individuals contributing feces or urine to the sewer samples [12–16]. Most wastewater utilities in the United States are governed by public utility commissions that serve public interests. However, to date, there have been no national assessments of sewer monitoring used for public health surveillance to determine public acceptance or concerns.

Applications that use impersonal data for service purposes, such as civil status (birth, death, and marriage), housing, elections, or work, are less likely to raise privacy concerns [17]. In contrast, technologies that use personal data for surveillance purposes, such as police data or images captured by closed-circuit television cameras, are more likely to raise privacy concerns [17]. In this regard, there are three recurring dimensions: sensitivity/personalness of the data, purpose (service versus surveillance) of data collection, and collector/user of the data [17]. Each dimension can be extended to sewer monitoring. For example, legislation opposing COVID-19 sewer monitoring includes North Dakota’s House Bill 1348, which was aimed at “prohibiting the testing of wastewater for genetic material or evidence of disease; and to provide a penalty,” did not pass in February 2021 [18]. Media reports focused on the privacy concerns of building-level surveillance and stated that the practice could violate college students’ privacy rights [19]. During the COVID-19 pandemic, the shifting policies of social restrictions determined by community infection levels created circumstances that raised concerns about sewer monitoring data being used as partial evidence for changing societal conduct, such as temporary closure conditions for communities, schools, or industries, as being credible.

Although WBE has been established for assessing public health inquiries [1–7], the COVID-19 pandemic has expanded the field and may have increased public awareness accordingly. Using a survey distributed to a sample of adults across the United States, the public perceptions related to sewer monitoring as a public health surveillance tool was investigated. This study aimed to assess (1) knowledge, awareness, and acceptance of sewer monitoring, (2) perceptions of privacy issues, and (3) factors that influence an individual’s level of awareness and acceptance of sewer monitoring. This study provides valuable insights into the acceptability of monitoring and informing development of policies regarding future applications of sewer monitoring at both national and local levels.

## Methods and materials

Survey invitations were sent to a randomly selected population who were enrolled in the research participant panels managed by Qualtrics XM (Provo, UT, USA). To limit selection bias, the invitation sent by Qualtrics XM advertised a short 15-min survey that was available to people who likely met the inclusion criteria. The inclusion criteria were tested using Qualtrics XM survey logic and data management methods, and were limited to English-speaking United States residents who were 18 years or older and did not live in rural communities. Invitations were sent in January and February 2022.

Participants who accepted the invitation were directed to a secure website, where they were provided a preamble informed consent. The completion of the survey, which included no identifiable or protected information, was considered an assent to participate. All participants were compensated by Qualtrics XM for their participation according to their panel participation agreement. The typical incentive value was $10.

### Data collection instrument

The 80-item survey (S1 File) included three components: (1) questions to assess knowledge, awareness, and acceptance of sewer monitoring; (2) questions covering demographics (gender identity, race, ethnicity, age, income, education level, and geography); and (3) questions on privacy concerns using the privacy attitude questionnaire (PAQ) [20]. The survey items comprised Likert-type scales (arranged in matrices), rank ordering, select one, and choose all.

The PAQ is used to structure privacy as a psychometric construct and considers privacy to be limited in both the physical and digital public environment centered on the four privacy attitudes: exposure (e.g., I would like to keep photos of my family on the internet), monitoring (e.g., I prefer to not have my name listed on a building directory), protection (e.g., I would prefer people to knock before coming into my office or bedroom), and willingness to share personal information (e.g., I am comfortable with giving a DNA sample). The PAQ measure used Likert scales (1–5, 5 = strongly agree), clustered into four factors of 8–10 items each. The PAQ used in this study was not specific to sewer monitoring but rather to assess the general privacy boundaries of respondents.

Several survey items were clustered into subscales. The reliability analyses revealed that the survey was interpreted and used as intended. The subscales included *knowledge of public health activities* (n = 6, α = 0.86), *support for sewage monitoring of activities* (n = 10, α = 0.87), *support for monitoring of locations* (n = 7, α = 0.84), *opposition to the monitoring of location types* (n = 6, α = 0.84), and the *PAQ* (n = 36, α = 0.60). The reliability estimates for the four PAQ domains were *exposure* (n = 9, α = 0.54), *monitoring* (n = 9, α = 0.39), *protection* (n = 8, α = 0.69), and *personal information* (n = 10, α = 0.70).

Additional items assessed *knowledge* (n = 3), *self versus other orientation* (n = 6), and *confidence and willingness to share personal information* (n = 6). The responses to these three subscales showed reliability estimates that were less acceptable (α values < 0.5).

Demographic data (n = 8) were also collected.

### Data management and analysis

Online surveys using participant panels pose the risk of false responses (e.g., providing random answers to earn an incentive), and to counter this, the survey was administered via Qualtrics XM to the participants who were regularly screened for fidelity. Qualtrics XM randomly distributes the survey invitations to its pool of participants who meet the inclusion criteria, performs an initial scrubbing of the data, and compensates participants whose data are deemed acceptable by the standards set by Qualtrics XM. These standards include satisfying a CAPTCHA test to access the survey, the time spent for completing the survey, missing data analysis, answer variability, and additional proprietary algorithms. Qualtrics XM prohibited respondents from completing the survey more than once. Data that did not meet Qualtrics XM quality control standards were excluded. The data collection resulted in 386 (11%) rejected responses. Two items were embedded in the survey as attention checks, and 100% of the respondents included in the analysis accurately answered both the attention checks.

**Fig 1:**
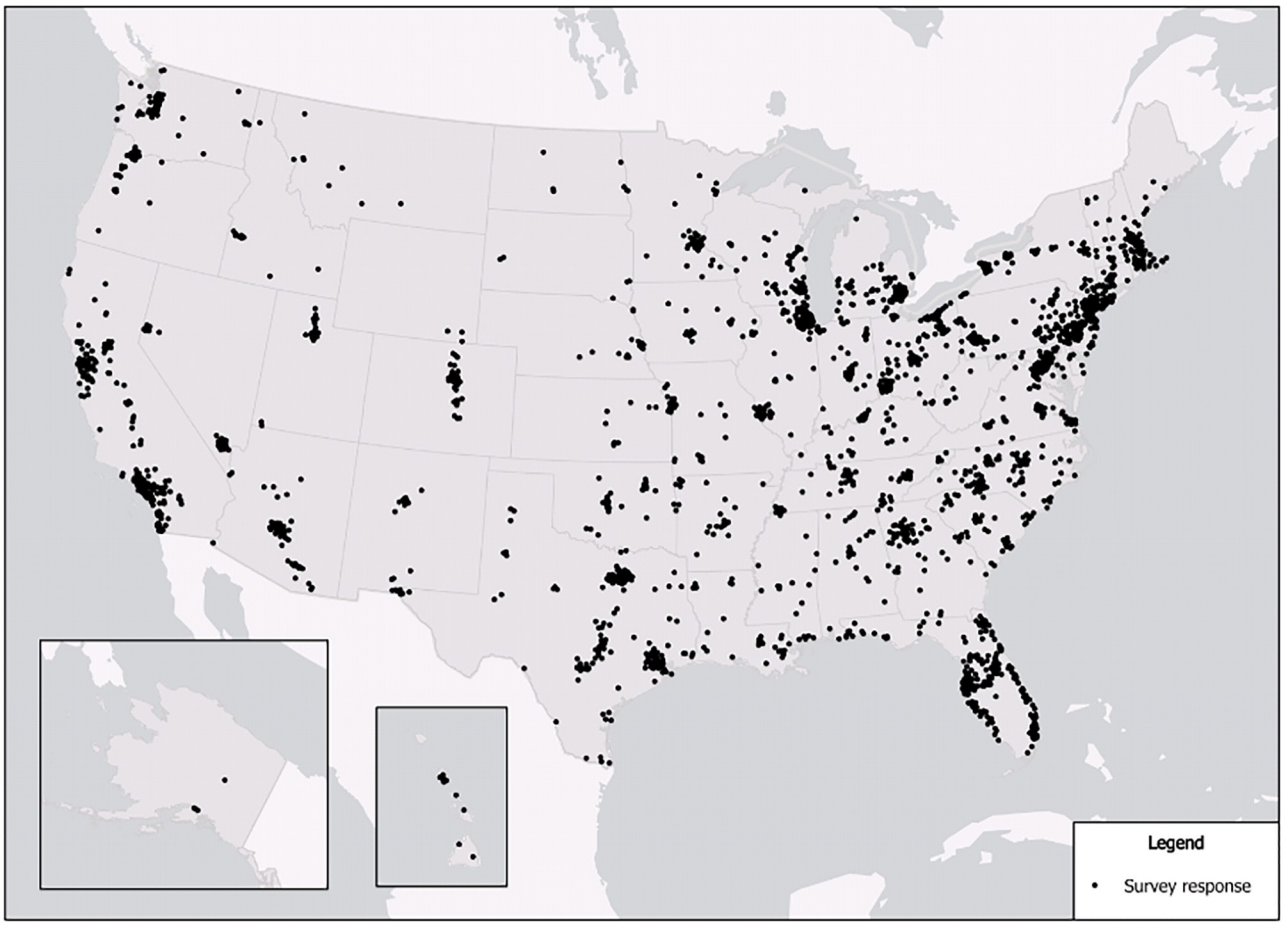
Location of respondents.

As most data were categorical, the analyses were largely restricted to descriptive measures and non-parametric tests, including frequency counts, cross-tabulations, chi-square, or Fisher’s exact tests. Pseudo-continuous variables were created where appropriate (i.e., subscales using the same measure type and having a Cronbach’s alpha value of > 0.6). The final sample size included 3,083 respondents from across the United States (Fig 1), with an estimated margin of error of ± 2%.

Statistical significance was set at an alpha value of <0.05. Data analysis was performed using the IBM Statistical Package for the Social Sciences (SPSS) software (version 28; Armonk, NY, USA) [21].

### Ethics

The University of Louisville Institutional Review Board approved this project as Human Subjects Research (IRB number: 21.0877).

## Results

Complete responses were obtained from 3,083 people (S2 File). The respondents were predominantly female (69%), white (84%), non-Hispanic (94%), and older than 54 years (66%). The income distribution skewed toward higher-income brackets, with approximately 25% having incomes between $20,000 and $40,000, 28% having incomes between $40,000 and $70,000, and 32% having incomes greater than $70,000. The sample was predominantly well educated, with many (79%) having some college education or beyond. More respondents lived in suburban areas (70%) rather than urban areas (30%). Table 1 provides a complete description of the sample.

**Table 1:** Demographic characteristics of respondents.

	N	Percent
**Gender**		
Female	2122	68.8%
Male	946	30.7%
Non-binary/third gender	11	0.4%
Prefer not to say	4	0.1%
**Age (years)**		
18–24	80	2.6%
25–34	234	7.6%
35–44	365	11.8%
45–54	375	12.2%
55–64	663	21.5%
65–74	1014	32.9%
75–84	323	10.5%
85 or older	29	0.9%
**Education**		
Less than High School	74	2.4%
High School graduate	566	18.4%
Some college	810	26.3%
2-year degree	392	12.7%
4-year degree	791	25.7%
Professional degree	402	13.0%
Doctorate	48	1.6%
**Income**		
Less than $10,000	164	5.3%
$10,000—$19,999	277	9.0%
$20,000—$29,999	401	13.0%
$30,000—$39,999	379	12.3%
$40,000—$49,999	338	11.0%
$50,000—$59,999	300	9.7%
$60,000—$69,999	236	7.7%
$70,000—$79,999	213	6.9%
$80,000—$89,999	150	4.9%
$90,000—$99,999	142	4.6%
$100,000—$149,999	307	10.0%
More than $150,000	176	5.7%
**How would you describe where you live?**		
Mostly urban	926	30.0%
Mostly suburban	2157	70.0%
**Race**		
White	2584	83.8%
Black	254	8.2%
American Indian/Alaska Native	16	0.5%
Asian	71	2.3%
Native Hawaiian and Pacific Islander	6	0.2%
Other	69	2.2%
Multiple Races	83	2.7%
**Hispanic**		
Yes	171	5.5%
No	2912	94.5%

### Descriptive findings

#### Basic knowledge of sewer monitoring of SARS-CoV-2

Three items assessed rudimentary knowledge of sewer monitoring for SARS-CoV-2 by asking similar questions in different styles. First, participants were asked whether COVID-19 could be detected in sewage. The correct answer (true) was selected by 42% of the participants, incorrect answer (false) was selected by 9%, and 48% said that they did not know. Next, respondents identified which, if any, of the five statements were false across a range of where and what to monitor in sewage. The correct answer (false), that monitoring sewage could determine which person or persons in a household had COVID-19, was selected by less than 50% of the respondents (48%). Finally, the respondents were asked to identify the fastest way to detect COVID-19 in a community. The correct answer, “measure the level of the virus in the sewer water,” was selected by 38% of the respondents, followed by "test everyone in the community (37%). A summary knowledge score was created from these three questions, with respondents earning one point for each correct answer. Possible knowledge scores ranged between 0 and 3, with 0 indicating no or limited knowledge and 3 indicating basic knowledge of sewage monitoring. The distribution of the respondents’ knowledge scores was roughly uniform: 0 (28%), 1 (32%), 2 (23%), or 3 (17%). The mean (standard deviation) was 1.28 (1.05). The respondents tended to have limited knowledge of the basic functions of sewage monitoring for SARS-CoV-2.

#### Awareness of public health surveillance functions

Respondents rated their awareness of six public health surveillance functions on a scale of 0 (no awareness) to 4 (full awareness) (Table 2). Participants were mostly aware (aware/fully aware) of restaurant inspections (87%), hotel and motel inspections (76%), drinking water quality testing (68%), public pool inspections (67%), but less aware of air pollution (50%) or sewer (49%) monitoring. The mean level of awareness across the six functions was 2.8. The respondents were less aware of sewer monitoring than other public health monitoring.

**Table 2:** Awareness and support (N = 3,083) by age, race, income, education, gender, and location.

	Age Bracket			Race (Dichotomized)		Income Bracket			Education			Gender (Dichotomized)		Residency		Overall
	18 to 44	45 to 65	65+	White	People of Color	Less than $40k	$40k to $79K	$80k or More	High School	College	Graduate School	Male	Female	Urban	Suburban	Total
	679	1038	1366	2584	499	1221	1087	775	640	1993	450	945	2122	926	2157	3083
Air pollution monitoring	286	488	776	1282	268	567	541	442	240	1049	261	559	980	470	1080	1550
	42%	47%	57%	50%	54%	46%	50%	57%	38%	53%	58%	59%	46%	51%	50%	50%
Drinking water testing	404	693	1000	1776	321	785	743	569	382	1378	337	691	1394	596	1501	2097
	59%	67%	73%	69%	64%	64%	68%	73%	60%	69%	75%	73%	66%	64%	70%	68%
Hotel and motel inspections	502	783	1072	2001	356	923	837	597	477	1523	357	710	1633	682	1675	2357
	74%	75%	78%	77%	71%	76%	77%	77%	75%	76%	79%	75%	77%	74%	78%	76%
Public pool inspections	409	668	981	1750	308	777	729	552	391	1348	319	638	1408	602	1456	2058
	60%	64%	72%	68%	62%	64%	67%	71%	61%	68%	71%	68%	66%	65%	68%	67%
Sewer monitoring	289	489	734	1261	251	556	548	408	270	997	245	522	980	445	1067	1512
	43%	47%	54%	49%	50%	46%	50%	53%	42%	50%	54%	55%	46%	48%	49%	49%
Restaurant inspections	555	908	1212	2269	406	1040	945	690	535	1743	397	813	1847	786	1889	2675
	82%	87%	89%	88%	81%	85%	87%	89%	84%	87%	88%	86%	87%	85%	88%	87%
Alcohol	298	458	683	1202	237	559	538	342	300	946	193	382	1054	422	1017	1439
	44%	44%	50%	47%	47%	46%	49%	44%	47%	47%	43%	40%	50%	46%	47%	47%
Deadly diseases (e.g., Ebola, Tuberculosis)	582	952	1300	2404	430	1099	1000	735	555	1857	422	867	1953	830	2004	2834
	86%	92%	95%	93%	86%	90%	92%	95%	87%	93%	94%	92%	92%	90%	93%	92%
Environmental toxins (e.g., industrial chemicals)	572	946	1301	2392	429	1090	999	732	546	1852	423	863	1944	822	1999	2821
	84%	91%	95%	93%	86%	89%	92%	94%	85%	93%	94%	91%	92%	89%	93%	92%
Gun residue (e.g., bullet casings, gun powder)	397	562	825	1479	305	714	639	431	382	1148	254	497	1279	549	1235	1784
	58%	54%	60%	57%	61%	58%	59%	56%	60%	58%	56%	53%	60%	59%	57%	58%
Healthy eating	297	309	357	732	231	417	341	205	238	602	123	264	696	348	615	963
	44%	30%	26%	28%	46%	34%	31%	26%	37%	30%	27%	28%	33%	38%	29%	31%
Illegal drugs	374	683	1008	1767	298	766	769	530	407	1351	307	624	1435	576	1489	2065
	55%	66%	74%	68%	60%	63%	71%	68%	64%	68%	68%	66%	68%	62%	69%	67%
Lifestyle behaviors (e.g., smoking, birth control)	281	311	347	725	214	393	343	203	229	589	124	261	677	325	614	939
	41%	30%	25%	28%	43%	32%	32%	26%	36%	30%	28%	28%	32%	35%	28%	30%
Mental illness (e.g., stress hormones)	347	425	485	1001	266	511	462	294	310	790	167	381	880	411	856	1267
	51%	41%	36%	39%	53%	42%	43%	38%	48%	40%	37%	40%	41%	44%	40%	41%
Prescription drugs	407	637	954	1663	335	778	730	490	405	1319	274	592	1399	597	1401	1998
	60%	61%	70%	64%	67%	64%	67%	63%	63%	66%	61%	63%	66%	64%	65%	65%
Terroristic threats (e.g., Anthrax)	545	902	1258	2293	412	1042	966	697	518	1784	403	831	1861	787	1918	2705
	80%	87%	92%	89%	83%	85%	89%	90%	81%	90%	90%	88%	88%	85%	89%	88%
Want monitored: Entire city	513	773	1062	1954	394	950	825	573	505	1506	337	703	1633	733	1615	2348
	76%	74%	78%	76%	79%	78%	76%	74%	79%	76%	75%	74%	77%	79%	75%	76%
Want monitored: Areas of the city	166	202	248	483	133	245	214	157	119	393	104	184	429	184	432	616
	24%	19%	18%	19%	27%	20%	20%	20%	19%	20%	23%	19%	20%	20%	20%	20%
Want monitored: Neighborhoods	183	242	295	569	151	316	239	165	163	449	108	209	508	232	488	720
	27%	23%	22%	22%	30%	26%	22%	21%	25%	23%	24%	22%	24%	25%	23%	23%
Want monitored: Businesses	143	182	216	410	131	232	193	116	118	355	68	146	393	173	368	541
	21%	18%	16%	16%	26%	19%	18%	15%	18%	18%	15%	15%	19%	19%	17%	18%
Want monitored: Prisons	166	206	255	483	144	279	212	136	137	402	88	185	440	202	425	627
	24%	20%	19%	19%	29%	23%	20%	18%	21%	20%	20%	20%	21%	22%	20%	20%
Want monitored: Schools	208	265	312	617	168	342	263	180	186	500	99	213	569	250	535	785
	31%	26%	23%	24%	34%	28%	24%	23%	29%	25%	22%	23%	27%	27%	25%	25%
Want monitored: Houses	132	169	168	349	120	216	154	99	129	290	50	118	349	161	308	469
	19%	16%	12%	14%	24%	18%	14%	13%	20%	15%	11%	12%	16%	17%	14%	15%
"I would support monitoring of all these places"	431	699	948	1726	352	860	735	483	463	1337	278	607	1460	660	1418	2078
	63%	67%	69%	67%	71%	70%	68%	62%	72%	67%	62%	64%	69%	71%	66%	67%
Want prohibited: Apartment buildings	93	130	119	293	49	135	121	86	58	230	54	112	228	89	253	342
	14%	13%	9%	11%	10%	11%	11%	11%	9%	12%	12%	12%	11%	10%	12%	11%
Want prohibited: Individual houses	205	292	338	715	120	298	288	249	142	547	146	274	557	214	621	835
	30%	28%	25%	28%	24%	24%	26%	32%	22%	27%	32%	29%	26%	23%	29%	27%
Want prohibited: K-12 schools, colleges, and universities	65	82	78	190	35	85	82	58	43	144	38	63	159	64	161	225
	10%	8%	6%	7%	7%	7%	8%	7%	7%	7%	8%	7%	7%	7%	7%	7%
Want prohibited: Nursing homes, assisted living facilities	66	84	76	199	27	82	89	55	46	147	33	70	154	59	167	226
	10%	8%	6%	8%	5%	7%	8%	7%	7%	7%	7%	7%	7%	6%	8%	7%
Want prohibited: Religious organizations	85	137	172	338	56	147	149	98	72	252	70	145	247	110	284	394
	13%	13%	13%	13%	11%	12%	14%	13%	11%	13%	16%	15%	12%	12%	13%	13%
Want prohibited: Rest areas and truck stops	54	78	99	201	30	85	81	65	39	155	37	76	153	63	168	231
	8%	8%	7%	8%	6%	7%	7%	8%	6%	8%	8%	8%	7%	7%	8%	7%
"I would not support any monitoring of sewage water"	69	131	126	271	55	122	122	82	66	213	47	96	226	76	250	326
	10%	13%	9%	10%	11%	10%	11%	11%	10%	11%	10%	10%	11%	8%	12%	11%

#### Support for sewer monitoring

When asked how strongly they would support or oppose sewer monitoring among ten indicators of human activity or health, many respondents were indifferent, opposed, or strongly opposed to monitoring lifestyle behaviors (70%; e.g., opposed monitoring of smoking, use of birth control), alcohol consumption (53%), diet (69%), and indicators of mental illness (59%; e.g., stress hormones). Fewer respondents were opposed or strongly opposed to the monitoring of illicit (33%) or prescription drugs (35%), and gun residue (42%). Finally, participants were most likely to support the monitoring of diseases (91%), environmental toxins (92%), and terrorist threats (87%; e.g., anthrax). Table 2 indicates the percentages of respondents supporting or strongly supporting these indicators of human activity or health.

Overall, on a scale of 1 to 5, where 5 is strongly supported, the mean level of support across these indicators was 3.7. People tended to be less supportive of monitoring self-controlled threats to health and more supportive of monitoring external threats.

#### Scale and location of monitoring

Respondents were asked if they would *want* or *prohibit* monitoring at different geographic scales (e.g., houses or entire city) and specific types of locations (e.g., prisons and college campuses) (Table 2). The intent was to elicit preferences regarding how diffuse or concentrated monitoring activities should be. Items were presented as a check-all. Approximately 90% of respondents agreed that, at the very least, certain areas should be monitored. Specifically, 76% of respondents wanted the entire city to be monitored. If not the entire city, respondents wanted schools (25%), neighborhoods (23%), and prisons (20%) to be monitored. Less support was evident in defined areas of cities (an area larger than neighborhoods but smaller than the entire city) (20%), businesses (18%), or houses (15%).

Respondents next indicated which types of locations they would prohibit monitoring (e.g., houses of worship, assisted living facilities, truck stops, and rest stops), with an option to alternatively support the monitoring of all categories. More respondents (67%) endorsed no prohibition on monitoring the locations. Other respondents considered that monitoring should be prohibited for individual households (27%), houses of worship and/or religious organizations (13%), and apartment buildings (11%). Some respondents wanted to prohibit from monitoring truck stops and rest areas (7%), school campuses (7%; e.g., K-12 and colleges), and nursing homes or assisted living facilities (7%). These results support other findings from this survey that monitoring at the individual or small cluster-level is less preferred; however, in general, respondents felt that monitoring should not be prohibited.

#### Factors for support or opposition to monitoring

Several items explored why respondents may be reluctant to monitor locations that could yield findings that are potentially specific at the individual-level. As sewer monitoring has the potential to gather data people may prefer to keep private, respondents were asked if they were confident that “city officials” could maintain the privacy of the collected information. Respondents rated their confidence in city officials in keeping personal information private. The three information types evaluated were health/medical, lifestyle/behaviors, and financial, with ratings ranging from 0 (no confidence) to 4 (complete confidence). Seventy-nine percent were confident or very confident that city or town leaders would keep all these types of information confidential. Lifestyle and behavioral information were the areas in which the highest percentage of the respondents were unsure or lacked confidence (18%), followed by financial information (17%) and health/medical information (16%).

When asked if they would be willing to give up privacy (none to all, on a scale of 0 to 4) to ensure that people in the community could live safe and healthy lives, 78% of the respondents reported being willing to give up some or all privacy related to these three information types, with the willingness to give up financial information related privacy being the least frequently endorsed one (55%). These results indicated high confidence in local government officials and only minor privacy concerns.

#### Privacy attitude questionnaire

**Fig 2:**
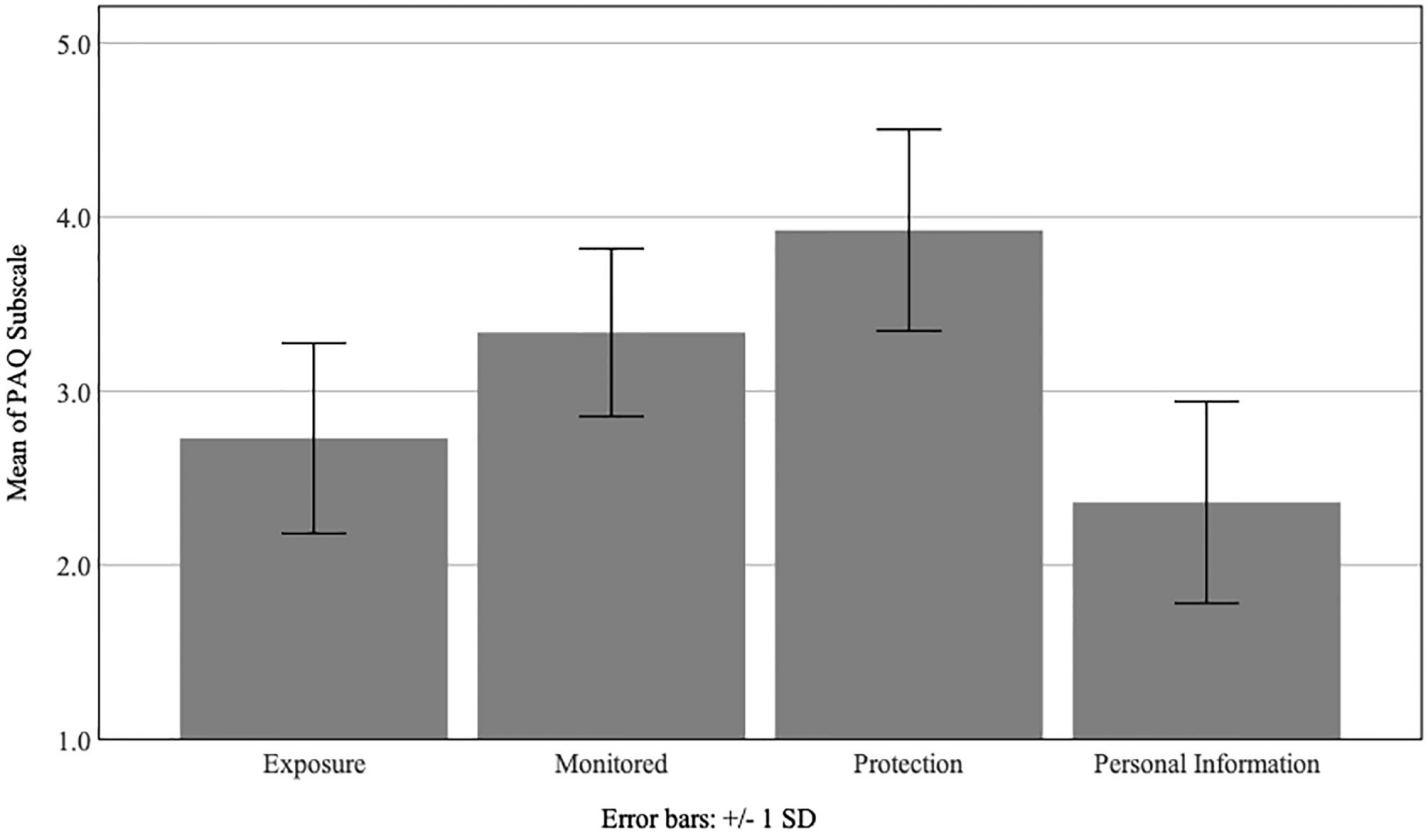
Responses from the privacy attitude uestionnaire for *exposure* (n = 9), *monitoring* (n = 9), *protection* (n = 8), and *personal information* (n = 10). A lower score indicates a higher level of privacy concern within each factor; error bars represent ± 1 standard deviation.

The PAQ (Fig 2) scores assess the willingness to disclose information that one might want to keep private. Aggregate mean (standard deviation) scores of the four scales were as follows: *exposure* = 2.73 (0.55), *monitoring* = 3.34 (0.48), *protection* = 3.92 (0.58), and *personal information* = 2.36 (0.58). Low scores indicated less willingness to disclose or a higher desire to remain private. Although respondents were more concerned about sharing their personal information than the other three factors, the aggregate mean PAQ score was consistent with confidence that “city officials” could maintain the privacy of the collected information.

### Inferential findings

Subgroups of respondents were analyzed to explore the differences in awareness, knowledge, and preferences for public health monitoring. Predictors included gender, race, age cohort, education level, income bracket, and urban/rural residency. Gender was limited to males and females, as there were too few respondents in the other gender identities to make for a separate category. For analyses including gender, the sample size reduced to 3067. Race was dichotomized into White or People of Color.

Tests of normality, including the Kolmogorov–Smirnov test and visual inspection of the Normal Q–Q plots, indicated that the three measures did not meet the criteria for univariate analysis of variance. Consequently, the Kruskal–Wallis *H* test was used to test for between-group differences. Post-hoc comparisons, when needed, were compared against the Behamini–Hockberg-adjusted *p-value* (*B-H p-value*). The *B–H p-value* is a conservative measure of the false discovery rate [22, 23].

#### Knowledge

The knowledge score (summation score ranging from 0 to 3, with 3 indicating higher knowledge) was tested for differences associated with the predictor variables. Statistical differences were found for **race** (white, *M* = 1.33 [*SD* = 1.06]; people of color, *M* = 1.01 [*SD* = 0.96]; *H* = 37.30, *df* = 1, *B-H p-value* < 0.001), **age cohort** (youngest as 18 to 44 years, *M* = 1.10 [*SD* = 1.01]; middle as 45 to 64 years, *M* = 1.25 [*SD* = 1.06]; oldest as 65+ years, *M* = 1.40 [*SD* = 1.05]; *H* = 38.45, *df* = 2, *B-H p-value <* 0.001), **schooling** (high school, *M* = 0.95 [*SD* = 0.98]; college, *M* = 1.31 [*SD* = 1.05]; graduate school, *M* = 1.61 [*SD* = 1.04]; *H* = 110.90, *df* = 2, *B-H p-value <* 0.001), **income bracket** (lowest as earning less than $40,000 annually, *M* = 1.14 [*SD* = 1.04]; middle as earning $40,000–$80,000 annually, *M* = 1.33 [*SD* = 1.04]; highest as earning $80,000 or more annually, *M* = 1.44 [*SD* = 1.05]; *H* = 41.78, *df* = 2, *B-H p-value <* 0.001), **gender** (males, *M* = 1.34 [*SD* = 1.04]; females, *M* = 1.26 [*SD* = 1.06]; *H* = 4.19, *df* = 1, *B-H p-value* = 0.04), and **residency** (urban, *M* = 1.18 [*SD* = 1.05]; suburban, *M* = 1.33 [*SD* = 1.05]; *H* = 15.17, *df* = 1, *B-H p-value* = 0.001). Those with higher knowledge included older white people who attended graduate school, reported higher incomes, were male, and lived in a mostly suburban area.

#### Awareness

The Kruskal–Wallis *H* statistic was used to test whether the average level of awareness of the six public health surveillance activities differed across different demographic variables. The mean score ranged from 0 (no awareness) to 4 (full awareness).

Statistical difference was found for **gender** (males, *M* = 2.89 [*SD* = 0.91]; females, *M* = 2.75 [*SD* = 0.94]; *H* = 15.36, *df* = 1, *p* < 0.001); **age cohort** (youngest, *M* = 2.64 [*SD* = 0.97]; middle, *M* = 2.77 [*SD* = 0.93]; oldest, *M* = 2.90 [*SD* = 0.92]; *H* = 42.42, *df* = 2, *B-H p-value <* 0.001); **schooling** (high school, *M* = 2.60 [*SD* = 0.99]; college, *M* = 2.83 [*SD* = 0.93]; graduate school, *M* = 2.93 [*SD* = 0.88]; *H* = 27.99, *df* = 2, *B-H p-value <* 0.001), and, **income bracket** (lowest, *M* = 2.73 [*SD* = 0.97]; middle, *M* = 2.81 [*SD* = 0.94]; highest, *M* = 2.89 [*SD* = 0.87]; *H* = 10.68, *df* = 2, *B-H p-value* = 0.005).

Awareness did not differ by **residency** (urban, *M* = 2.74 [*SD* = 1.00]; suburban, *M* = 2.82 [*SD* = 0.91]; *H* = 2.15, *df* = 1, *B-H p-value* = 0.14) or **race** (white, *M* = 2.81 [*SD* = 0.93]; people of color, *M* = 2.74 [*SD* = 1.00]; *H* = 1.57, *df* = 1, *B-H p-value* = 0.21). Those with higher awareness of the six public health surveillance activities were male, older, had more education, and had higher incomes.

#### Support for the monitoring of ten activities

Respondents indicated their level of support for the monitoring of ten activities: use of illicit drugs, prescription drugs, alcohol, eating habits, lifestyle behaviors, gun residue, mental illness, toxins, terrorist threats, and diseases. Responses ranged from 1 (strongly opposed) to 5 (strongly supported). An aggregate measure of support was created by averaging the ten potentially monitorable activities; the closer it was to 5, the higher the support. The Kruskal–Wallis *H* statistic was used to test for differences in support among the various independent variables.

The average level of support differed by **gender** (males, *M* = 3.65 [*SD* = 0.75]; females, *M* = 3.76 [*SD* = 0.75]; *H* = 15.45, *df* = 1, *B-H p-value* < 0.001).

**Age** cohort did not influence the mean level support (youngest, *M* = 3.69 [*SD* = 0.79]; middle, *M* = 3.70 [*SD* = 0.77]; oldest, *M* = 3.77 [*SD* = 0.71]; *H* = 6.70, *df* = 2, *B-H p-value* = 0.16). The **income** bracket did not influence the mean level of support (lowest, *M* = 3.69 [*SD* = 0.79]; middle, *M* = 3.70 [*SD* = 0.77]; highest, *M* = 3.76 [*SD* = 0.75]; *H* = 4.17, *df* = 2, *B-H p-value* = 0.13), and neither did **residency** (urban, *M* = 3.73 [*SD* = 0.76]; suburban, *M* = 3.72 [*SD* = 0.76]; *H* = 0.005, *df* = 1, *B-H p-value* = 0.94), nor **education** (high school, *M* = 3.74 [*SD* = 0.76]; college, *M* = 3.73 [*SD* = 0.74]; graduate school, *M* = 3.68 [*SD* = 0.78]; *H* = 2.27, *df* = 2, *B-H p-value* = 0.32).

In general, females tended to show higher support for monitoring the ten activities than males.

#### Support for monitoring of the entire city

The endorsement of monitoring the entire city was not associated with gender, race, age, income, or education. However, those living in urban areas endorsed monitoring the entire city at a higher percentage than those living in suburban areas (79% versus 74%; Fisher’s exact test = 0.006).

#### Supporting the monitoring of all places

Respondents were asked if they would prohibit the monitoring of certain places, such as apartment buildings; alternatively, they were given the option to support monitoring of "all of these places." Demographic comparisons were made for the option, support the monitoring of these places. **Gender** was associated with support for monitoring. Female respondents (69%) were more likely than male respondents (64%) to select not to prohibit any location (Fisher’s exact test = 0.007). **Age cohort** was associated with support for monitoring (Chi-square [*df* = 2] = 7.25, *p* = 0.027); the youngest cohort was less likely (63%) to select “support all” compared to the middle (67%) and older (69%) cohorts. **Education** level was associated with selecting to "support all" (Chi-square [*df* = 2] = 13.68, *p* = 0.001); those with a high school education (72%) were more likely to select “support all” than those with at least some college (67%) or graduate school education (62%). **Income bracket** was associated with selecting to "support all" for monitoring (Chi-square [*df* = 2] = 14.23, *p <* 0.001); those in the lowest income bracket were more likely to make this selection (70%) compared to the middle-income (68%) and the highest income (62%) brackets. **Residency** was associated with the selection to prohibit no location from monitoring (Fisher’s exact test = 0.001). Urban dwelling respondents (71%) were more likely than suburban respondents (66%) to support monitoring. There was no association between race and selecting "support all" locations for monitoring (white [67%] versus people of color [71%]; Fisher’s exact test = 0.11).

Thus, respondents who were female, older, less educated, less wealthy, and living in urban areas tended to endorse supporting all locations for monitoring. Conversely, younger, more educated, wealthier, suburban males were more likely to endorse prohibiting at least some places for monitoring.

#### Predicting support for the monitoring of specific public health targets

A linear regression equation was constructed using a stepwise approach to understand the demographic characteristics that predicted the support for sewer monitoring of ten activities (use of illicit drugs, prescription drugs, alcohol, eating habits, lifestyle behaviors, gun residue, mental illness, toxins, terrorist threats, and diseases). The predictor variables were entered into the model as follows: mean score of awareness of public health surveillance functions; the four PAQ mean subscale scores (exposure, monitoring, protection, and personal information); and the six demographic variables: gender (1 = male, 2 = female), age cohort (1 = youngest, 2 = middle, 3 = oldest), race (0 = people of color, 1 = white), income (1 = lowest, 2 = middle, 3 = highest), residency (1 = urban, 2 = suburban), and education (1 = high school, 2 = some college, 3 = graduate school).

A significant regression equation was obtained (*F*(6, 3066) = 58.32, *p <* 0.001), with an *R*^*2*^ = 0.103 for the strength of support for monitoring the 10 activities.

The three of the four PAQ subscales, gender, and age cohort were significant predictors (*p*s < 0.05); PAQ-exposure, race, education, income bracket, and residency were excluded (*p*s > 0.05). The respondents predicted that the *Support of Ten Activities* score was equal to 1.26 + PAQ-monitoring (0.35) + awareness (0.09) + PAQ-protection (0.13) + PAQ-personal information (0.10) + gender (0.11) + age cohort (0.03). Thus, positive attitudes toward disclosing private information, female gender, and older age tended to predict increasing strength toward support for monitoring sewage for certain activities.

## Discussion

In this study, a nationally distributed public opinion survey was used to assess the knowledge, awareness, and privacy concerns regarding sewer monitoring and individual factors. Approximately half of the respondents did not know whether COVID-19 could be detected in sewage. Further, we found that the awareness of sewer monitoring across the United States was generally lower than that of other types of public health surveillance.

Overall, respondents tended to show moderate levels of privacy concerns, as measured by the PAQ, with higher levels of concern about personal information and less levels of concern when disclosure was associated with protection. Overall, respondents supported sewer monitoring, particularly for identifying risks to health, such as terrorist threats, toxins, and diseases. However, less support was offered for studying the behaviors and health status of the population, such as monitoring lifestyle behaviors, healthy eating, and mental illness.

The use of sewers to monitor community health status leads to salient tradeoffs. Croft et al. [3] studied illicit and prescribed neuropsychiatric drugs in sewers, uniquely spanning choice activities and mental health issues. Assessing mental health through sewer monitoring, using stress hormones [24] as a quantitative measure, offers an opportunity to highlight needs and bring more advocacy to low-income or other communities that may struggle with social and environmental justice. However, the privacy concerns of individuals versus a community should be balanced with the real and valid concerns that sewer monitoring could be used as a tool for surveilling and administering punishment or stigmatization to a community. For example, identifying evidence of illicit drug use within sewers could result in negative outcomes for neighborhoods, such as increased policing and negative media attention.

Our respondents more often supported surveillance at the most anonymous levels, such as citywide pooled samples, over smaller-scale sampling. The results are consistent with the guidelines of Hall et al. [12] and Scassa, Robinson, and Mosoff [16], which suggest that community sewer monitoring is generally ethically acceptable; however, when monitoring is conducted on small scales, such as workplaces, prisons, and schools, it may elicit increased concerns. Our national survey results also correspond with those of an earlier study that focused on views within Louisville, Kentucky, which showed increased public support for sewer measurements in the largest areas possible (> 50,000 households) [25].

Determining the scope and context of monitoring and clarifying public benefits and privacy protection is perhaps a precursor for rule-making. North Dakota’s proposed ban for sewer monitoring via House Bill 1348 [18] suggest that privacy concerns around sewer surveillance exist and that monitoring is open to public debate. These debates can be complex. For example, the monitoring of alcohol consumption was supported by about half of the respondents in our study and studies such as Ryu et al. [5] have additionally shown that sewer monitoring allows consumption rates and weekly patterns of drinking to be calculated. However, because the United States federal minimum drinking age is 21 years, age of the subjects’ excretion to the piped sewer network could be a key to future privacy protection and public debate.

Knowledge, awareness, and support for sewer monitoring varied across the respondents, and this variance could be partially explained by education, income, gender, and age. Race and residence generally had smaller effects. For example, younger people with lower levels of education are nearly as supportive of monitoring as their older and more educated peers; however, but they tended to have lower awareness and knowledge to strengthen this support. Our survey also complements a survey by Hill et al. [26], who showed that wastewater utility supervisors had some knowledge of sampling sewers for public health; with targeted education programming evidenced on the limitations and benefits of sampling still considered necessary for this group. In wastewater reuse, negative public opinion has been found to be driven by pathogen disgust [27]. Therefore, one recommendation following this national survey is to use science communication to improve public knowledge and awareness of this health surveillance tool. Education and outreach tools, such as websites and data dashboards, should be widely available and provide jargon-free and accessible explanations of the sewer monitoring being conducted.

The limitations of sewer monitoring applications regarding privacy for public health should be acknowledged; they are best established when utilizing existing piped sewer system infrastructure. This type of infrastructure covers approximately 85% of the United States population [28] residing mostly in urban and suburban areas, thereby allowing a degree of anonymity with homogeneous sewer samples from many individuals. Our results show public support for monitoring in these areas. However, the remaining 15% of the United States population [28] resides within either dominantly rural areas containing lagoon systems, septic tanks, and straight pipes or as outlier higher-income households with large landholdings away from urban centers. These rural places have less individual household privacy in the sewer monitoring approach than the urban dwellers. Respondents seem to intuitively know this and thus, more frequently indicated that individual systems should be prohibited from monitoring. However, some rural areas offer valuable public health opportunities for surveillance, such as mobile home communities and congregate care facilities. These locations typically have poor communication with local health departments, and sewer monitoring can provide early warnings.

In early 2022, the Centers for Disease Control and Prevention launched the National Wastewater Surveillance System [29] providing further support for this technology. The initial version of this platform is accessible to the public; however, it is limited to SARS-CoV-2 data to date. As the field of sewer monitoring continues to build capacity, utility providers, public health practitioners, and environmental health practitioners must engage with the public to balance the need for public health monitoring with privacy protection.

## Limitations

While the invitations to participate were randomly sent to a large, diverse population, participants were self-selected to participate. The invitation itself did not reveal the nature of the survey until the participant met the inclusion criteria. However, as is the case with all panel survey methods, self-selection bias may exist among the pool of people interested in survey research. The completion of the survey was skewed toward respondents who were older, wealthier, better educated, and suburban dwelling. The inclusion criteria excluded rural dwelling individuals. Finally, the results focused on the United States, and further research is required to gather public perceptions regarding the acceptance of the use of sewers for global community health monitoring.

## Conclusions

Using an online survey of English-speaking, non-rural adults in the United States, we investigated public perceptions regarding what should be monitored, where monitoring should occur, and privacy concerns related to sewer monitoring as a public health surveillance tool. The incorporation of sewer surveillance into broad use has been propelled by the COVID-19 pandemic; however, it remains unfamiliar to a large percentage of the public in the United States. The most important finding of this study may be the absence of significant nationwide concerns regarding privacy violations from sewer monitoring. A second important finding is that sampling at the scale of an entire city was supported more than that at the scale of smaller areas. Lastly, sewer monitoring is an emerging technology, and this study highlights some perceptions that could benefit from educational programs in areas where acceptance or concern about public health surveillance is comparatively low. The results of our study show that although knowledge and awareness of sewer monitoring was low, respondents clearly communicated guard rails for what is and is not acceptable for monitoring, and public opinion should inform future policy, application, and regulation measures of wastewater-based epidemiology practices.

## Supporting information

S1 File This is the data collection instrument. (PDF)

S2 File This is the data in a supporting information file. (XLSX)
