# Supplementary material for: Nationwide public perceptions regarding the acceptance of using wastewater for community health monitoring in the United States
Source: PLoS One. 2022 Oct 11;17(10):e0275075. doi: 10.1371/journal.pone.0275075 (PMC9553059; doi:10.1371/journal.pone.0275075)
Supplement: S1 File — (PDF) [file pone.0275075.s001.pdf]

UNIVERSITY OF LOUISVILLE

CENTER FOR HEALTHY AIR, WATER AND SOIL

Pre-Amble

You are being invited to participate in a national research study by answering questions about monitoring sewage water to understand potential sources of harm (e.g., disease) in a community. This study is being conducted by Drs. Ted Smith and Scott LaJoie of the University of Louisville.

There are no known risks of participating. The information collected may not benefit you directly. The information learned in this study may be helpful to others. The information you provide will help us to develop recommendations and guidelines for future public health strategies that use community-based sewage monitoring.

Your survey answers will be stored in a secure computer in an office at the School of Public Health and Information Sciences at the University of Louisville. The survey includes 58 questions and will take approximately 13 minutes.

Individuals from the School of Public Health and Information Sciences, the Envirome Institute, the University of Louisville, the

Institutional Review Board (IRB), the Human Subjects Protection Program Office (HSPPO), and other regulatory agencies may inspect these records. In all other respects, the data will be held in confidence to the extent permitted by law. Should the data be published, your identity will not be disclosed.

Taking part in this survey is voluntary. By answering survey questions, you agree to participate in this research study. You may choose to not participate at all. You do not have to answer any questions that make you uncomfortable. You may stop at any time. If you decide to stop participating, you will not lose any benefits for which you may qualify.

If you have any questions, concerns, or complaints about the research study, please contact: Dr. Ted Smith, 502-852-3993 (Principal Investigator) Dr. Scott LaJoie, 502-852-1879 (Principal Investigator)

If you have any questions about your rights as a research subject, you may call the Human Subjects Protection Program Office at (502) 852-5188. You can discuss any questions about your rights as a research subject, in private, with a member of the Institutional Review Board (IRB). The IRB is an independent committee made up of people from the University community, staff of the institutions, as well as people from the community not connected with these institutions. The IRB has reviewed this research study. If you

have any questions, concerns, or complaints about the research or research staff and you do not wish to give your name, you may call 1-877-852-1167. This is a 24-hour hotline answered by people who do not work at the University of Louisville.

Sincerely,

Ted Smith & Scott LaJoie

Screeners/Demographics

What gender do you identify as?

- ○ Male
- ○ Female
- ○ Non-binary / third gender
- ○ Prefer not to say
- ○ [ ] Other

What is your age range?

- ○ Under 18
- ○ 18 - 24

- ○ 25 - 34
- ○ 35 - 44
- ○ 45 - 54
- ○ 55 - 64
- ○ 65 - 74
- ○ 75 - 84
- ○ 85 or older

What is your race? You may choose more than one option.

- ☐ White
- ☐ Black or African American
- ☐ American Indian or Alaska Native
- ☐ Asian
- ☐ Native Hawaiian or Pacific Islander
- ☐ ______ Other

Are you Hispanic?

- ○ Yes
- ○ No

What is the highest level of education you completed?

- Less than high school
- High school graduate
- Some college
- 2 year degree
- 4 year degree
- Professional degree
- Doctorate

What is your household income?

- Less than $10,000
- $10,000 - $19,999
- $20,000 - $29,999
- $30,000 - $39,999
- $40,000 - $49,999
- $50,000 - $59,999
- $60,000 - $69,999
- $70,000 - $79,999
- $80,000 - $89,999
- $90,000 - $99,999
- $100,000 - $149,999
- More than $150,000

How would you describe where you live?

- Mostly urban

○ Mostly suburban

○ Mostly rural

What is your 5-digit zip code?

[ ]

Default Question Block

The Health Department and other public health agencies conduct environmental activities to protect the public's well-being. Many people are not aware of these activities, while some are fully aware. For each activity, select the circle to show your level of <u>awareness</u> (0 = No Awareness, 4 = Full Awareness).

	0 (No awareness)	1	2	3	4 (Full awareness)
Air Pollution Monitoring	○	○	○	○	○
Drinking Water Testing	○	○	○	○	○
Sewage Water Monitoring	○	○	○	○	○

The Health Department and other public health agencies conduct facilities activities to protect the public's well-being. Many people are not aware of these activities, while some are fully aware. For each activity, select the circle to show your level of awareness (0 = No Awareness, 4 = Full Awareness).

	0 (No awareness)	1	2	3	4 (Full awareness)
Restaurant Inspections	○	○	○	○	○
Hotel and Motel Inspections	○	○	○	○	○
Public Pool Inspections	○	○	○	○	○

If it were available, would you support or oppose the City monitoring sewage water for the following items? For each activity, select the circle to show your level of support or opposition.

	Strongly oppose	Oppose	Indifferent	Support	Strongly support
Illegal drugs	○	○	○	○	○
Prescription drugs	○	○	○	○	○
Alcohol	○	○	○	○	○

If it were possible, would you support or oppose the City monitoring sewage water for the following items? For each activity, select the circle to show your level of support or opposition.

	Strongly oppose	Oppose	Indifferent	Support	Strongly support
Terroristic threats (e.g., Anthrax)	○	○	○	○	○
Environmental toxins (e.g., industrial chemicals)	○	○	○	○	○
Deadly diseases (e.g., Ebola, Tuberculosis)	○	○	○	○	○
Gun residue (e.g., bullet casings, gun powder)	○	○	○	○	○

If it were possible, would you support or oppose the City monitoring sewage water for the following items? For each activity, select the circle to show your level of support or opposition.

	Strongly oppose	Oppose	Indifferent	Support	Strongly support
Mental illness (e.g., stress hormones)	○	○	○	○	○
Lifestyle behaviors (e.g., smoking, birth control)	○	○	○	○	○

	Strongly oppose	Oppose	Indifferent	Support	Strongly support
Healthy eating	○	○	○	○	○

Scientists have found that the SARS-2-CoV virus, which causes COVID-19, can be detected in the feces of people who have COVID-19. Is it true or false that the virus can be detected in the sewage waters?

- ○ False
- ○ True
- ○ I don't know

If Public Health wanted to learn the percentage of people in a community who are infected with a virus, like COVID-19, what would be the fastest way to do it?

- ○ Test everyone in the community
- ○ Count the number of infected people in the Emergency Department
- ○ Measure the level of the virus in the sewer water
- ○ Conduct a survey to ask people if they are sick
- ○ Ask employers how many people call in sick in the past week

Which statement, if any, is False? Select one.

- ○ Coronavirus can be detected in sewage water for up to two weeks
- ○ Monitoring sewage water can show if new strains of COVID-19 are present
- ○ Coronavirus can be detected in the sewage water of a single neighborhood
- ○ Coronavirus can be detected in the sewage water of a single building, like a hospital, business, or school
- ○ Monitoring sewage water can determine which person or persons in a household has COVID-19
- ○ None of these statements are false

Block 1

If the city were to monitor sewage water, what would you want monitored? Check all that apply

- ☐ The entire city
- ☐ Areas of the city
- ☐ Neighborhoods
- ☐ Businesses
- ☐ Prisons and jails
- ☐ Schools
- ☐ Houses
- ☐ I would not support any monitoring of sewage water

Which of the following, if any, should the City be prohibited from sewage monitoring? Check all that apply.

- ☐ Religious organizations
- ☐ K-12 Schools, Colleges and Universities
- ☐ Nursing homes, assisted living facilities
- ☐ Individual households
- ☐ Rest areas and truck stops
- ☐ Apartment buildings
- ☐ I would support monitoring of all of these places

There are numerous areas where sewer monitoring does not apply. For example, sewer monitoring does not work with septic tanks. Among the following options, select the option where sewer monitoring does not work.

- ○ Public sewer systems
- ○ City run drainage
- ○ Septic tanks

Block 2

Drag to arrange the options to show what is most important to you. Your first option will be the one 'most important' and the last option will be 'least important'.

Protect the health of my loved ones

Protect the health of people I know

Protect the health of people I do not know

Protect the health of all people in my city

Protect my health

Block 3

For each statement, select the circle to rate how much the statement describes how you think about yourself. (0 = Not well at all, 4 = Extremely well)

	0 (Not well at all)	1	2	3	4 (Extremely well)
I depend on myself more that I depend on others	○	○	○	○	○
I will do extra effort if it helps out someone else	○	○	○	○	○
I will help others only if I get 'credit' for doing so	○	○	○	○	○
I will pay extra taxes if it helps people who are less fortunate than I am	○	○	○	○	○

For each statement, select the circle to rate how much the statement describes how you think about yourself. (0 = Not well at all, 4 = Extremely well)

	0 (Not well at all)	1	2	3	4 (Extremely well)
I am happy for other people when they get an award, even if I did not	○	○	○	○	○
Protect myself and my family is more important than protecting other people	○	○	○	○	○

Block 4

How much confidence do you have in City Officials to keep your personal information private? (0 = No confidence, 4 = Complete confidence)

	0 (No confidence)	1	2	3	4 (Complete confidence)
Health or Medical information	○	○	○	○	○
Financial information	○	○	○	○	○
Lifestyle / behaviors information	○	○	○	○	○

How much of your privacy would you be willing to give up to ensure that people in your community can live safe and healthy lives? For each activity, select the circle to show your level of willingness (0 = None at all, 4 = All of it)

	0 (None at all)	1	2	3	4 (All of it)
Health or Medical information	○	○	○	○	○
Financial information	○	○	○	○	○
Lifestyle / behaviors information	○	○	○	○	○

Block 6

I would like to keep photos of my family on the internet.

Strongly disagree	Somewhat disagree	Neither agree nor disagree	Somewhat agree	Strongly agree
○	○	○	○	○

I'd object to my photograph appearing in a public place without my permission.

Strongly disagree	Somewhat disagree	Neither agree nor disagree	Somewhat agree	Strongly agree
○	○	○	○	○

I would put my photo on my personal web page.

Strongly disagree	Somewhat disagree	Neither agree nor disagree	Somewhat agree	Strongly agree
○	○	○	○	○

I would like to participate in reality TV.

Strongly disagree	Somewhat disagree	Neither agree nor disagree	Somewhat agree	Strongly agree
○	○	○	○	○

I would like a high fence in my backyard.

Strongly disagree	Somewhat disagree	Neither agree nor disagree	Somewhat agree	Strongly agree
○	○	○	○	○

No organization or person should disseminate personal information about me without my knowledge.

Strongly disagree	Somewhat disagree	Neither agree nor disagree	Somewhat agree	Strongly agree
○	○	○	○	○

I tell some of my work colleagues about my personal life.

Strongly disagree			Somewhat agree	Strongly agree

	Somewhat disagree	Neither agree nor disagree		
○	○	○	○	○

I like to tell stories about my family to my friends.

Strongly disagree	Somewhat disagree	Neither agree nor disagree	Somewhat agree	Strongly agree
○	○	○	○	○

I would not mind appearing on television or being quoted in a newspaper.

Strongly disagree	Somewhat disagree	Neither agree nor disagree	Somewhat agree	Strongly agree
○	○	○	○	○

I frequently would like to block my phone number on call display.

Strongly disagree	Somewhat disagree	Neither agree nor disagree	Somewhat agree	Strongly agree
○	○	○	○	○

I respond to telephone marketing surveys.

Strongly disagree	Somewhat disagree	Neither agree nor disagree	Somewhat agree	Strongly agree
○	○	○	○	○

I prefer not to have my name listed on a building directory.

Strongly disagree | Somewhat disagree | Neither agree nor disagree | Somewhat agree | Strongly agree

Video cameras should be used in public places to improve public safety and security.

Strongly disagree | Somewhat disagree | Neither agree nor disagree | Somewhat agree | Strongly agree

I would give my home phone number to business clients

Strongly disagree | Somewhat disagree | Neither agree nor disagree | Somewhat agree | Strongly agree

I am not paying close attention to these items.

Strongly disagree | Somewhat disagree | Neither agree nor disagree | Somewhat agree | Strongly agree

I like to fill out surveys and contests.

Strongly disagree	Somewhat disagree	Neither agree nor disagree	Somewhat agree	Strongly agree
○	○	○	○	○

Red light (intersection) cameras should be used.

Strongly disagree	Somewhat disagree	Neither agree nor disagree	Somewhat agree	Strongly agree
○	○	○	○	○

Speeding cameras should be used.

Strongly disagree	Somewhat disagree	Neither agree nor disagree	Somewhat agree	Strongly agree
○	○	○	○	○

Insurance companies should not have access to people's health records.

Strongly disagree	Somewhat disagree	Neither agree nor disagree	Somewhat agree	Strongly agree
○	○	○	○	○

I would prefer to have an unlisted phone number.

Strongly disagree	Somewhat disagree	Neither agree nor disagree	Somewhat agree	Strongly agree
○	○	○	○	○

I like to clear my cache (from visiting websites online) frequently for privacy reasons.

Strongly disagree	Somewhat disagree	Neither agree nor disagree	Somewhat agree	Strongly agree
○	○	○	○	○

I like to change my passwords frequently.

Strongly disagree	Somewhat disagree	Neither agree nor disagree	Somewhat agree	Strongly agree
○	○	○	○	○

I like to close my curtains at home at night.

Strongly disagree	Somewhat disagree	Neither agree nor disagree	Somewhat agree	Strongly agree
○	○	○	○	○

I frequently question why I'm providing personal information.

Strongly disagree	Somewhat disagree	Neither agree nor disagree	Somewhat agree	Strongly agree
○	○	○	○	○

I would prefer people to knock before coming into my office or bedroom.

Strongly disagree	Somewhat disagree	Neither agree nor disagree	Somewhat agree	Strongly agree
○	○	○	○	○

I worry about the possibility that my conversations will be overheard.

Strongly disagree	Somewhat disagree	Neither agree nor disagree	Somewhat agree	Strongly agree
○	○	○	○	○

I would use a personal firewall on my computer.

Strongly disagree	Somewhat disagree	Neither agree nor disagree	Somewhat agree	Strongly agree
○	○	○	○	○

I am comfortable with giving a DNA sample.

Strongly disagree	Somewhat disagree	Neither agree nor disagree	Somewhat agree	Strongly agree
○	○	○	○	○

I am comfortable giving out my personal identification number.

Strongly disagree	Somewhat disagree	Neither agree nor disagree	Somewhat agree	Strongly agree
○	○	○	○	○

I am comfortable in allowing others to check my credit.

Strongly disagree	Somewhat disagree	Neither agree nor disagree	Somewhat agree	Strongly agree
○	○	○	○	○

I am comfortable wearing a name tag.

Strongly disagree	Somewhat disagree	Neither agree nor disagree	Somewhat agree	Strongly agree
○	○	○	○	○

Employers should be able to monitor employee email.

Strongly disagree	Somewhat disagree	Neither agree nor disagree	Somewhat agree	Strongly agree
○	○	○	○	○

It is ok to use messaging services even if the messages could in principle be tracked.

Strongly disagree	Somewhat disagree	Neither agree nor disagree	Somewhat agree	Strongly agree
○	○	○	○	○

I allow strangers to enter my house while I’m not there.

Strongly disagree	Somewhat disagree	Neither agree nor disagree	Somewhat agree	Strongly agree
○	○	○	○	○

I am comfortable with having my retina scanned.

Strongly disagree	Somewhat disagree	Neither agree nor disagree	Somewhat agree	Strongly agree
○	○	○	○	○

I do not mind using my real name in online discussions.

Strongly disagree	Somewhat disagree	Neither agree nor disagree	Somewhat agree	Strongly agree
○	○	○	○	○

My medical information should never be communicated to people or organizations without my permission.

Strongly disagree	Somewhat disagree	Neither agree nor disagree	Somewhat agree	Strongly agree
○	○	○	○	○
